# Automated Exploration of Free Energy Landscapes Based on Umbrella Integration

**DOI:** 10.3390/ijms19040937

**Published:** 2018-03-21

**Authors:** Yuki Mitsuta, Takashi Kawakami, Mitsutaka Okumura, Shusuke Yamanaka

**Affiliations:** Graduate School of Science, Osaka University, Osaka 565-0871, Japan; mitsutay13@chem.sci.osaka-u.ac.jp (Y.M.); kawakami@chem.sci.osaka-u.ac.jp (T.K.); ok@chem.sci.osaka-u.ac.jp (M.O.)

**Keywords:** automated search, potential of mean force, umbrella integration

## Abstract

We present a new approach for automated exploration of free energy landscapes on the basis of the umbrella integration (UI) method. The method to search points in the landscape relies on the normal distributions and gradients of the potential of mean force (PMF) obtained from UI calculations. We applied this approach to the alanine dipeptide in solution and demonstrated that the equilibrium and the transition states were efficiently found in the ascending order of the PMF values.

## 1. Introduction

Potential of mean force (PMF) is a central concept [1,2] for chemistry in condensed matter systems, which is given by

(1) $$A\left(\xi \right)=-k_{B}T\ln Q\left(\xi \right)$$

at constant temperature T (K), where *k_B_* is the Boltzmann constant and Q(ξ) is the effective partition function (or the configuration integral) of the canonical ensemble with fixing the number of particles (N). Q(ξ) is defined as,

(2) $$Q\left(\xi \right)=\frac{1}{h^{3N}N!}\int^{\text{​}}dr^{N}dp^{N}\delta \left(\hat{R}\left(r^{N}\right)-\xi \right)\exp\left[-H\left(r^{N},p^{N}\right)/k_{B}T\right]$$

Here, $H\left(r^{N},p^{N}\right)$ is the Hamiltonian of the target system and $\hat{R}\left(r^{N}\right)$ is the projection of the coordinate, ***r****^N^*, into the reaction coordinate. PMF can be considered as effective free energy in the reaction coordination because we can straightforwardly obtain Helmholtz free energy by integrating Q(ξ) over ξ. In fact, the profile of the PMF along the reaction coordination is often called the “free energy landscape” [1,2]. In actual calculations, molecular dynamics (MD) calculations are performed to yield the probability distribution for the reaction coordination,

(3) $$P\left(\xi \right)\propto \int^{\text{​}}dr^{N}dp^{N}\delta \left(\hat{R}\left(r^{N}\right)-\xi \right)\exp\left[-H\left(r^{N},p^{N}\right)/k_{B}T\right]\;\propto \int^{\text{​}}dr^{N}\delta \left(\hat{R}\left(r^{N}\right)-\xi \right)\exp\left[-V\left(r^{N}\right)/k_{B}T\right]$$

where *V*(***r****^N^*) is the potential term including inter-particle interactions in the Hamiltonian. Thus if we obtain the probability distribution, the PMF can be estimated via the relation,

(4) $$A\left(\xi \right)=-k_{B}T\ln P\left(\xi \right).$$

However, as is known, most chemical events such as chemical reactions and isomerization of molecules involve unstable and transient states, like transition states for which the sampling with straightforward MD calculations is difficult because the probability of the state specified by ξ exponentially decays as presented in the integral function at the rightest side of Equation (3). The theoretical researchers in this field have proposed various methods to prevent MD trajectories from being trapped in a local minimum and to sample “rare” states. One of such methods is the elaborate generalized ensemble approaches, such as the replica exchange method [3] and the multicanonical ensemble method [4,5]. As this type of method does not require to specify any reaction coordinate, it is useful for thoroughly searching global free energy minima, say, in protein holding problems, although it takes huge computational costs in general. Another class of methods, called thermodynamic integration [6,7] or blue moon sampling [8,9], is a more straightforward approach. In these types of methods, the free energy difference between $\xi_{1}$ and $\xi_{2}$ states, is written by,

(5) $$A\left(\xi_{1}\right)-A\left(\xi_{2}\right)=\int_{\xi_{1}}^{\xi_{2}}d\xi \frac{\partial A\left(\xi \right)}{\partial \xi }=\int_{\xi_{1}}^{\xi_{2}}d\xi {\langle \frac{\partial V\left(r^{N}\right)}{\partial \xi }\rangle }_{\xi }$$

Here, ${\langle \;\rangle }_{\xi }$ is the ensemble average over the coordinates for the fixing ξ, and so ${\langle \frac{\partial V\left(r^{N}\right)}{\partial \xi }\rangle }_{\xi }$ is a mean force along ξ. Equation (5) implies that the PMF can also be obtained via an integration of the mean forces over ξ, each of which is obtained with the MD calculation for a fixed ξ. There is another type of method, called “umbrella sampling (US)”, in which ξ is not fixed but restrained by adding a bias potential (or bias potentials) to sample the whole region along the reaction coordinate [10,11,12,13,14,15]. The standard strategy, termed “stratification”, among many versions of the US methods is to split the whole region of ξ into several windows and to use a bias potential to sample all the windows including rare states along ξ. Then, gathering the local probability histograms, the unbiased Boltzmann statistical probability is yielded by a statistical manipulation. A popular choice of the bias potential and the statistical manipulation are the harmonic bias and the weighted histogram analysis (WHAM) method [16], respectively, of which the combination, abbreviated as “US + WHAM” hereafter, is known as one of the standard stratified US methods, and is, hence, also employed in this study (see the following sections for details). The essential point of the stratified US method is how to determine windows and biases. A preferable choice is to cover the whole range along the reaction coordinate, ξ, in fewer windows (ideally one window) and to choose a bias, ω(ξ)=−A(ξ) to lead to a uniform sampling along ξ. An approach that achieves this goal is the adaptive biasing force (ABF) method [17,18,19], in which one estimates local biasing forces (not bias potentials) and updates those during the simulation run. Then, the PMF can be estimated with a thermodynamic integration given by Equation (5). In this context, metadynamics is a method similar to the ABF method, which aims at the same goal, i.e., achieving ω(ξ)=−A(ξ) and a uniform sampling. The different point is that the metadynamics method flattens the PMF surface with the bias (i.e., ω(ξ)=−A(ξ)) by dropping Gaussian functions along ξ during the simulation process to prevent the system from lingering in a specific local minimum [20,21]. For more details of ABF, metadynamics, and other related methods that use updating biases, see chapter 4 in ref. [2]. What we would like to emphasize here is that the convergence of the ABF and related methods relies on the nearly stochastic diffusion as the potentials are supposed to be nearly flattened during the simulation runs. As the dimension of the reaction coordinate increases, the computational time increases rapidly. 

Of course, the stratified US method with the predetermined bias potential such as the US + WHAM method also suffers from the same difficulty for high-dimensional problems if we intend to obtain the PMF over all the range of the reaction coordinates. What we need is an efficient and systematic exploration approach for many-dimensional problems. Recently, Kästner and his coworkers proposed the umbrella integration (UI) method, by which the gradients and the Hessians of the unbiased PMF, not the unbiased probability, are estimated [6,22,23,24,25]. This enables us to find minimum free-energy paths [25] without describing the whole landscape of the PMF. On the other hand, Wojtas-Niziurski et al. proposed an automated search method based on the US + WHAM method, by which they describe not only the minimum free-energy paths, but also the local PMF landscape using ongoing sampling data [14]. They exploit the local PMF starting from the US + WHAM calculation for several localized windows and extend the region to describe the PMF according to the free energy landscape obtained at the previous step. In fact, they showed that the essential characteristics of the PMF landscape can be described more efficiently than the usual US + WHAM method for both the conformational space of Met-enkephalin and ion permeation in the KcsA potassium channel. This approach is a powerful and useful method for automated search of the free energy surface. In this work, we employ a new automated approach, in which we exploit the gradients obtained with the umbrella integration method, instead of the local PMF obtained from the previous runs. The advantage of our method is that it is based on the gradients yielded by the UI method, which are expected to guide us to explore the landscape efficiently as in the case of the method to find minimum free-energy paths that was proposed by Kästner [25]. In this study, we formulate and implement our automated exploration approach based on the UI method. We applied the method to the free-energy landscape of the alanine dipeptide system and showed that, in fact, we can start from one point in the reaction coordinate and extend the explored region efficiently. We examined the efficiency of our method by comparing among the on-going free energy landscapes and those obtained with the usual US + WHAM scheme.

## 2. Theoretical Method

### 2.1. Umbrella Integration Method

First, we will briefly describe the umbrella integration (UI) method. The UI method uses bias potentials to sample relatively unstable regions in the free energy landscapes. Here, we employed a harmonic bias,

(6) $$\omega_{i}\left(\xi \right)=\frac{1}{2}{\left(\xi -\xi_{i}^{ref}\right)}^{t}K_{i}\left(\xi -\xi_{i}^{ref}\right)$$

for the MD calculation at each (*i*-th) window. Here, ξ is the reaction coordinate and $\xi_{i}^{ref}$ is the center of the bias potential. **K*_i_*** is the force constant matrix having off-diagonal (non-zero) elements in general. However, we assume that **K*_i_*** is a diagonal matrix throughout this study (see Equation (A11)). By the MD run for each window, we obtain the biased distribution, $P_{i}^{b}\left(\xi \right)$. Hereafter, we use the superscripts, “b” and “u”, for the properties which are, respectively, obtained from a simulation with the biased potential given by Equation (6) and processed with some manipulation to unbias the property. Using $P_{i}^{b}\left(\xi \right)$, we can calculate unbiased free energy,

(7) $$A_{i}^{u}\left(\xi \right)=-\frac{1}{\beta }\ln P_{i}^{b}\left(\xi \right)-\omega_{i}\left(\xi \right)+F_{i},$$

where *F_i_* is a window-dependent term. The weighted histogram analysis method (WHAM), which is a standard approach to obtain the unbiased free-energy from the calculated biased distributions, calculates {*F_i_*} iteratively by minimizing the statistical error of unbiased distribution [16]. 

In the umbrella integration (UI) scheme [22,23,24,25], we start from the gradient of the unbiased free energy that is straightforwardly obtained from Equation (7),

(8) $$\nabla A_{i}^{u}\left(\xi \right)=-\frac{1}{\beta }\nabla \ln P_{i}^{b}\left(\xi \right)-\nabla \omega_{i}\left(\xi \right),$$

An important point of the UI scheme is that the probability distribution for each window is approximated as a normal distribution,

(9) $$P_{i}^{b}\left(\xi \right)≅\frac{1}{{\left(2\pi \right)}^{n/2}{\left|C_{i}\right|}^{1/2}}exp\left[-\frac{1}{2}{\left(\xi -{\langle \xi \rangle }_{i}^{b}\right)}^{t}C_{i}^{-1}\left(\xi -{\langle \xi \rangle }_{i}^{b}\right)\right],$$

which is equivalent to the second order approximation of the cumulant expansion. Here, $C_{i}$ and ${\langle \xi \rangle }_{i}^{b}$ are the covariance matrix and the mean value (the average point) of ξ, respectively, which are obtained from a simulation for the *i*-th window. Substituting Equations (6) and (9) into Equation (8), we have

(10) $$g_{i}^{u}\left(\xi \right)\equiv \nabla A_{i}^{u}\left(\xi \right)=C_{i}^{-1}\frac{1}{\beta }\left(\xi -{\langle \xi \rangle }_{i}^{b}\right)-K_{i}\left(\xi -\xi_{ref}\right)$$

Note that this is the gradient of the free-energy obtained for the *i*-th window. In order to combine these gradients, we have to take a weighted average over windows as,

(11) $$\nabla A^{u}\left(\xi \right)\equiv g_{}^{u}\left(\xi \right)=\sum_{i}p_{i}\left(\xi \right)g_{i}^{u}\left(\xi \right)$$

Here, $p_{i}\left(\xi \right)$ is the normalized weight,

(12) $$p_{i}\left(\xi \right)=\frac{N_{i}P_{i}^{b}\left(\xi \right)}{\sum_{j}^{}N_{j}P_{j}^{b}\left(\xi \right)}$$

where *N_i_* (*N_j_*) is the number of sampling points for the *i*-th (*j*-th) window. The unbiased free-energy landscape, i.e., $A_{}^{u}\left(\xi \right)$ can be obtained by numerical integration of the gradients [13]. However, we should note that in our approach, the UI method is used only for searching the points in the landscape and the WHAM method is used for estimating $A_{}^{u}\left(\xi \right)$.

### 2.2. A New Automated Exploration Approach

In this section, we will describe a new automated exploration approach based on the umbrella integration scheme. The computational process starts from a specific window in the reaction coordinate space, where we perform an umbrella integration (UI) calculation described in the above section. If we have already many points that were already sampled and exist in the border of the sampled region, we choose the one which has the lowest PMF value. We then create new points around the sampled point and choose one among them for a new window. Throughout the procedure, two types of points appear, i.e., the point for which we already sampled and the point that is spawned from the sampled point. We call the former the “parent point” and the latter the “child point”, respectively. Once the child point is determined, we perform an UI calculation for the window. We prepare a list for the sampled windows, in which we store information of the distributions calculated for the windows. The list also includes information of whether or not each window exists on the border of the sampled region so that it is able to be a “parent point”. The procedure can be summarized as follows:

(0) Start from an umbrella integration (UI) calculation for a specific point in the reaction coordinate space.
(1) Choose a “parent point”, which has the lowest PMF value among the points that were already sampled and exist in the border region in the reaction coordinate space.
(2) Create new points around the parent point chosen in step (1).
(3) Prune the points created in step (2) and if all points are rejected, the parent point is judged to not be in the border region. Then go back to step (1).
(4) Choose the “child point” from among the points remaining in step (3).
(5) We perform an UI calculation of the new window for the child point chosen in step (4) and added information of the distribution for the window to the list of the windows. Then, go back to step (1).

The condition to terminate the computational process depends on the purpose of the computation. If we intend to cover the whole region of the reaction coordinate space, which is the case similar to the usual US + WHAM procedure, the computation is complete when we explore almost all regions of the space so that we cannot create points any further in step (2). In contrast, if we would like to explore the region where the free energy is lower than a threshold (to avoid to search the region that is unrealistic from the viewpoint of kinetics), we set a threshold of the free energy in advance, and we stop the computation process when we cannot find any point that has a free energy lower than the threshold in step (1). 

Especially in the latter case, the efficiency of the search strongly depends on where the first window is set at step (0). To avoid searching meaningless regions where there are no important equilibrium points and transition states, it is preferable to start from one of the lowest minima in the PMF landscape. There are several choices of the initial configuration. A simple choice is the optimized geometry of the target molecule(s) obtained either at molecular mechanics or at ab initio quantum mechanics level in vacuum. For almost all cases, this is an appropriate choice, because such optimized geometries are good starting points to one of the minima in the PMF landscape. If the thermal fluctuation is expected to considerably affect the stable geometries of the target molecule(s), we should perform a MD calculation of the molecule(s) with environmental molecules at the finite temperature and select the representative structure of the target molecule(s) from the trajectory. Of course, if there is a reliable equilibrium geometry reported in a previous study, we can start the geometry.

Next, we will describe the details of steps (1)–(5). For simplicity, we assume that the reactive coordinate space is two-dimensional in the following discussion. However, the algorithm can be straightforwardly extended to the multidimensional cases in general. 

**Figure 1:**
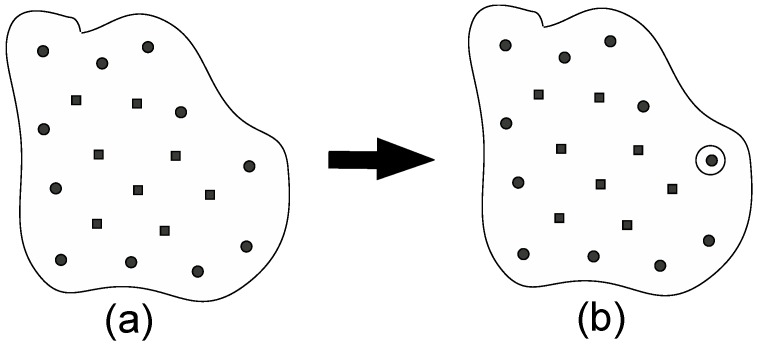
(**a**) Classification of sampled points in the reaction coordinate space. The black circles are the sampled points that exist in the border region. (**b**) The black circle point having the lowest PMF value is chosen as the parent point, around which new points will be created in step (2).

Figure 1 shows a schematic illustration of step (1). Now, we consider the case where there are many sampled points, which are shown as the black circles and the black squares. If there is only one sampled point just after step (0), step (1) is skipped. The black circles shown in Figure 1a are the positions of the averages (the centers) of the distribution calculated for the corresponding window that lie in the border region, while the black squares are also the sampled points but do not lie in the border region. How to judge whether the point lies in the border or not will be described in details in step (3). For every point, we have the temporal PMF values estimated with using WHAM. We then choose the point that has the lowest PMF value among these black circles, which is encircled by a solid circle, as shown in Figure 1b. This will become the parent point for step (2).

**Figure 2:**
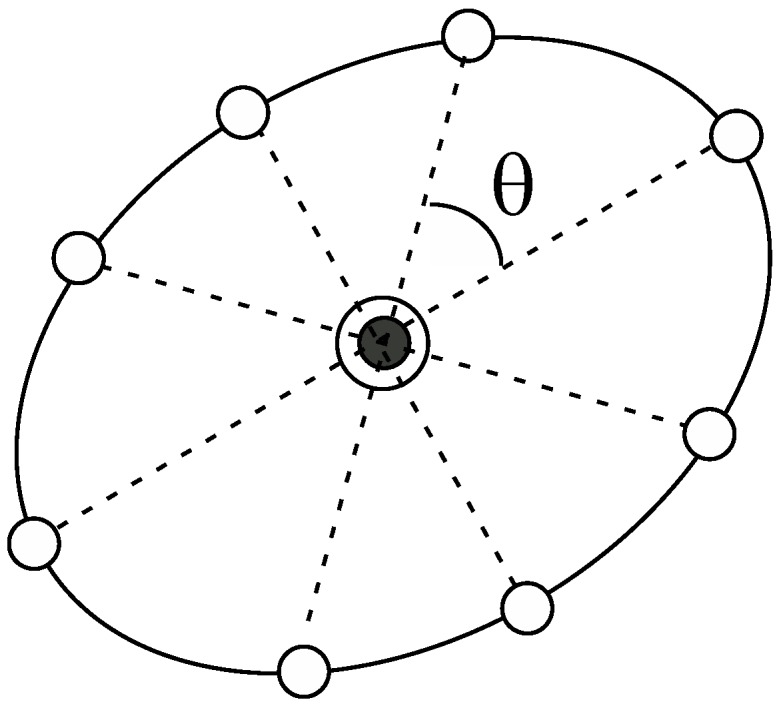
The ellipsoid that surrounds the parent point (the black circle enclosed by a solid circle). The equiangular points on the ellipsoid are the candidate points for the next windows.

Now, we proceed to step (2). At the present stage, we assume that a UI calculation has been already done for the parent point we choose in step (1). Using the resultant biased distribution, which can be rewritten by diagonalizing the matrix, **C**^−1^, as

(13) $$P_{i}^{b}\left(x\right)≅\frac{1}{{\left(2\pi \right)}^{n/2}{\left(\prod_{j}^{}{\left(\sigma_{j}^{i}\right)}^{2}\right)}^{1/2}}\exp\left[-\frac{1}{2}\sum_{j}\frac{{\left(x_{j}-{\langle \xi_{j}\rangle }_{i}^{b}\right)}^{t}\left(x_{j}-{\langle \xi_{j}\rangle }_{i}^{b}\right)}{\sigma_{j}^{2}}\right]$$

we form an ellipsoid, of which the center is placed on the average point of the distribution $P_{i}^{b}\left(\xi \right)$, i.e., ${\langle \xi \rangle }_{i}^{b}$, in order to set next windows. To explore the free-energy landscape efficiently, it would be better to rely on the metric based on the obtained distributions, not on the actual reaction coordinate, because the former reflects the feature of the landscape but the later does not. In the area around the center of the anisotropic normal distribution in the many dimensional space, the Mahalanobis metric [26] becomes an excellent measure to define the distance. Thus, we determined the directions of axes $\left\{e_{j}\right\}\;$ and the radiuses of the ellipsoid on the basis of the Mahalanobis metric of the multidimensional normal distribution, which are given by

(14) $$e_{j}=\frac{x_{j}}{{\left|x_{j}\right|}_{p}}$$

and

(15) $$R_{j}=3\sigma_{j}^{2},$$

respectively. Here, the notation, ${\left|x_{j}\right|}_{p}$ indicates the Mahalanobis distance of the vector $x_{j}$ based on the distribution of the parent window. In Figure 2, we show a schematic illustration of the ellipsoid that surrounds the parent point (the black circle enclosed by a solid circle). It is noteworthy that we multiply the variance, $\sigma_{j}^{2},$ by 3 to determine the radius, considerably reducing the number of windows in the following calculations. This does not deteriorate computational accuracy because, in the case of the umbrella integration, the dependency of the computational accuracy on the overlap between the distributions of the windows is not significant, in contrast to WHAM. In addition, Kästner and Thiel analyzed the error of the UI scheme and concluded that it is preferable to take the distance between the windows to be less than $3/\sqrt{\beta K}\sim 3\sigma_{j}^{2}$ for one-dimensional cases (**K** is the force constant of the harmonic oscillator) [20]. This is the reason why we introduce the guideline given by Equation (15). In fact, this choice of the radiuses practically leads to reasonable results as presented in the following calculations. We then create equiangular points (the white circles) on the ellipsoid as shown in Figure 2, which are the candidate points for the next windows; if the number of the points is N, θ is set to be 360°/N. For many dimensional cases, this procedure should be slightly changed. The details will be described in the Discussion and Future directions.

**Figure 3:**
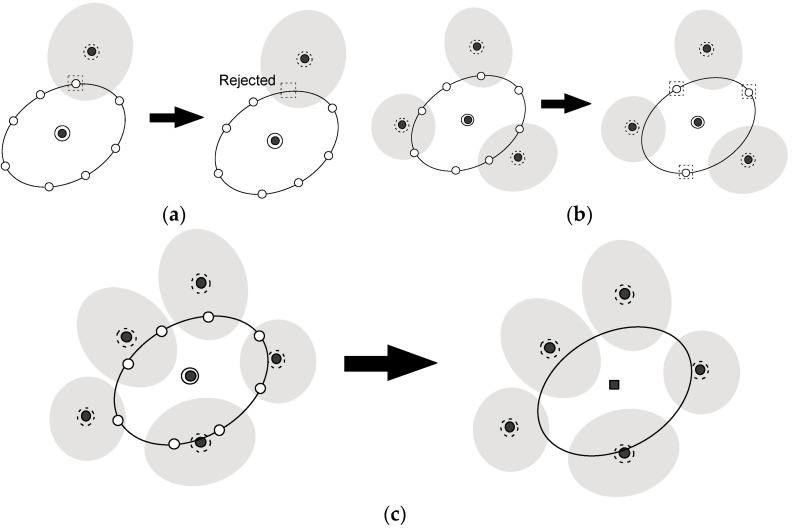
(**a**) If a candidate point is close enough to the sampled window, the candidate point will be rejected. For details, see text. (**b**) The pruning process in step (3): the white circles encircled by dotted squares remain as candidate points for the child point that will be determined in step (4). (**c**) The case that all the child points are rejected: we will go back to step (1) for this case.

As the exploration procedure proceeds, the region that is covered by the sampled region spreads out over the reaction coordinate space. Thus, in step (3), we have to prune the new (candidate) points created in step (2) if those are close enough to any of the sampled widows where we already performed simulations with the biased potentials. Let us consider the situation shown in Figure 3a,b. The problem is whether a new candidate point, ξ, should be pruned or not. To this end, we estimate the distributions at the candidate point,  ξ, for all neighboring windows that were already sampled, i.e., ${\left\{P_{i}^{b}\left(\xi \right)\right\}}_{i}^{all\;neighboring}$ and call the *k*-th window, of which $P_{k}^{b}\left(\xi \right)$ becomes maximum among all neighboring windows, the “nearest neighboring (N.N.) window”. Suppose that we would like to judge whether the point encircled by the dotted square should be pruned or not, and that the windows, of which the centers are encircled by the dotted circles shown in Figure 3a,b, is the N. N. window. Then, consider the region where the Mahalanobis distance between the center of the nearest neighboring (*k*-th) window and the candidate point, which is defined on the basis of the distribution of the N.N. window, $P_{k}^{b}\left(\xi \right)$, is less than 2.5. Such regions are shown as gray ellipsoids in Figure 3a,b. If a candidate point is within the region, it is supposed to be close enough to the sampled window (the *k*-th window of which the center is shown as the black circle enclosed by a dotted circle) and so is rejected as shown in Figure 3a. Otherwise, the points remain as candidate points for the child point (white circles encircled by dotted squares in Figure 3b). In this manner, we check all the candidate points. If all candidate points were rejected, the parent point is judged not to be in the border region as shown in Figure 3c, and go back to step (1). If there remain any candidate points, we proceed to step (4). 

**Figure 4:**
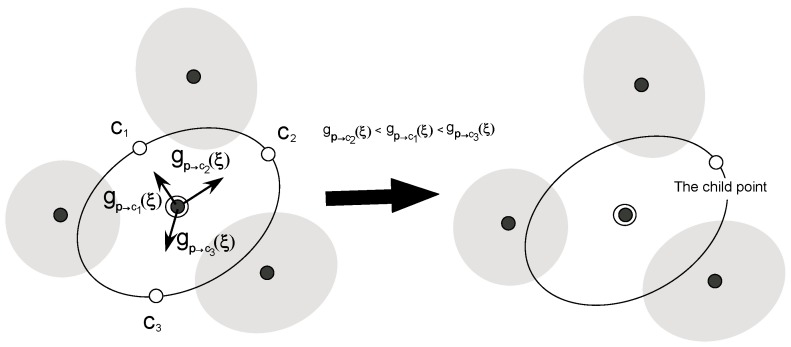
The determination step (step (4)) of the child point. First, we estimate the gradients along the directions from the parent point to candidate points as shown in the left side of the figure. Then, we choose the candidate point to which the direction is steepest descent as the child point as in the right side of this figure.

In step (4), we choose the child point from among the remaining candidate points. To explore lower free energy regions in the reaction coordinate space efficiently, we calculate $g_{p\to c_{i}}^{u}\left(\xi \right)=\nabla_{p\to c_{i}}A_{}^{u}\left(\xi \right)$ at the parent point using Equation (10), where $\nabla_{p\to c_{i}}$ is the gradient along the direction from the parent point to each candidate point, *c_i_*. Among all candidate points, the point to which the direction has the steepest descent is chosen as the child point, since the point is expected to have the lowest PMF value. For instance, if there are three candidate points, *c*_1_, *c*_2_, and *c*_3_, and the gradients are in the following order, $g_{p\to c_{2}}^{u}\left(\xi \right)<g_{p\to c_{1}}^{u}\left(\xi \right)<\;g_{p\to c_{3}}^{u}\left(\xi \right)$, then, *c*_2_ becomes the child point as shown in Figure 4. 

In step (5), we perform a UI calculation of the new window for the child point that we chose in step (4). At this stage, there still remains one problem. Even if we employ the child point as the center of the bias potential of Equation (6), $\xi_{c}^{ref}$, the center of the distribution of the UI calculation would drastically deviate from $\xi_{c}^{ref}$ with varying the force constant matrix, **K**, of the biased potential given by Equation (6). How to adjust **K** in order to make our algorithm work strongly depends on to what extent we accept the deviation between $\xi_{c}^{ref}$ and the center of the distribution of the UI calculation for the child window. Because the treatment involves a subtle but complicated procedure, the details will be presented in Appendix. Anyway, after the simulation run was performed using the parameter determined by the method described in the Appendix, the center of the distribution of the run, not the original child point, will be a candidate for a parent point for the next step. Thus, information of the window, together with the biased distribution obtained from the run, is stored in a list concerning the sampled windows. Then go back to step (1). 

It should be noted that the UI method is used only for spawning points to be sampled efficiently, and the WHAM method is used to estimate the free-energy landscape from the calculated windows at each number of iterations. This is because the UI method is prone to statistical error in estimating the free-energy landscape in higher dimensions, while the WHAM can be straightforwardly applied to multidimensional cases, as pointed out by Kästner [13]. 

The computational results will be presented in the next section.

## 3. Computational Results

**Figure 5:**
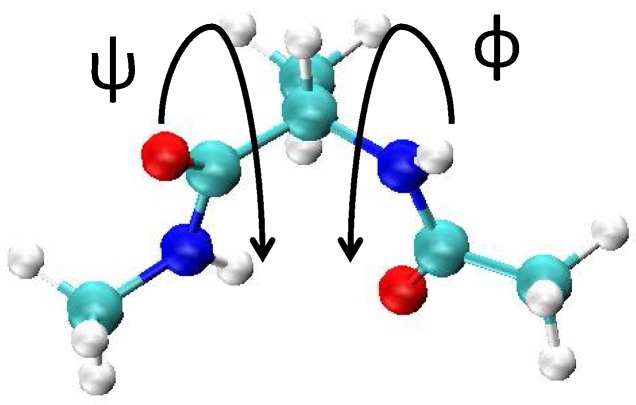
The structure of the alanine dipeptide we examined. The torsion angles, φ and ψ, that are used for the reaction coordinate are also shown.

Our method is applied to a simple and standard example, an alanine dipeptide in solution. We examined the free-energy landscape for the two backbone torsion angles, φ and ψ, shown in Figure 5. The alanine dipeptide was solvated in a cubic water box, which contains 354 water molecules, using the periodic boundary condition and the electrostatic interactions were treated using the particle-mesh Ewald method [27] with the cutoff of 10 Å. The CHARMM27 force field [28] and the TIP4P force field [29] were used for the alanine dipeptide and the water molecules, respectively. All the simulation runs were performed with the time step of 2 fs under the constant NPT condition, for which T was kept constant at 298 K using the V-rescale thermostat [30] and P at 1 bar using the Berendsen barostat [31]. The LINK method is used to constrain the bonds [32]. For each window, the system was equilibrated for 50 ps and then simulated for 1 ns to obtain the distribution. All the MD runs were performed using GROMACS 5.1.4 [33] and PLUMED 2.3.1 [34]. For the WHAM calculations, we used the code developed by Grossfield [35].

**Figure 6:**
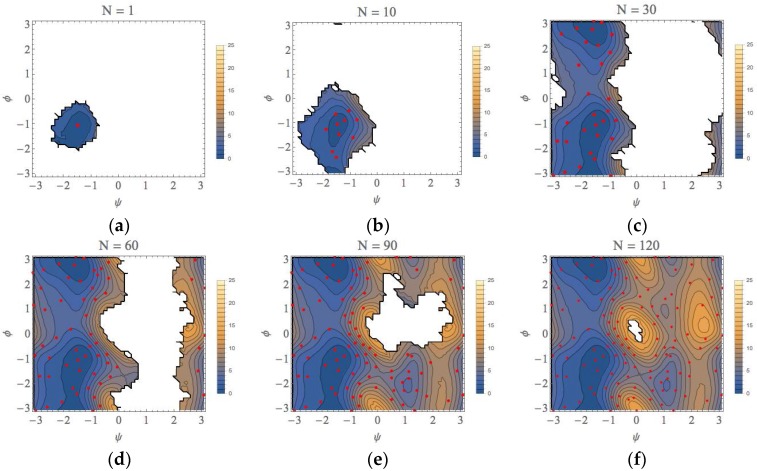
The free-energy landscapes of the (φ, ψ) space for the alanine dipeptide system with using our exploration scheme. N is the number of iterations to obtain each figure. (**a**) N = 1, (**b**) N = 10, (**c**) N = 30, (**d**) N = 60, (**e**) N = 90, (**f**) N = 130.

For an initial umbrella integration (UI) run, the center of the bias potential is set at the point (ψ, φ) = (−1.5, −1.0) (unit: radian). The geometry of this point was taken from a previous study of the conformational search of the alanine dipeptide in explicit water using an umbrella sampling calculation [36]. This point is in the basin of the α_R_ conformation that was confirmed to be most stable among four types of conformations [36]. We set that the force constant of the bias potential for the MD runs is 100 kJ/mol/rad^2^ for both of ψ and φ directions. Starting from the distribution obtained from this first run, we applied the automated exploration scheme described above to explore the free energy landscape. The results for the selected number of iterations are shown in Figure 6 (more comprehensive data are shown in Figure S1). In these figures, the average points (parent points) determined by our approach were plotted as red points and the contours in the landscape are colored as indicated by the right bar (unit: kcal/mol). At the first iteration, the parent point of the first UI run was the most stable point of the α_R_ conformation shown Figure 6a, but, as the number of iterations (N) increased, the most stable point of the α_R_ conformation shifted to (ψ, φ) = (−1.4, 1.1). It is noteworthy that not only the minimum point, but also the local free energy landscape for the α_R_ conformation was correctly determined until N = 10, as shown in Figure 6b. Then, the exploration procedure reached at the C_7^eq^_ conformation ((ψ, φ)~(−1.5, 2.9)) at N = 23 (Figure S1 (23)) and the PMF difference between the α_R_ and C_7^eq^_ conformations is estimated to be less than 1 kcal/mol, which is quite similar to the final result, 0.0 kcal/mol (N = 132). As shown in Figure 6c (see also Figure S1 (23)–(30)), the local basin of the C_7^eq^_ conformation has been almost characterized until N = 30. The landscapes of the C_7^ex^_ conformation and the α_L_ conformation were characterized at N~90 and N~120, respectively, as shown in Figure 6e,f. This is because our automated exploration procedure finds preferentially the points with lower PMF values. The representative structures for all the conformations are shown in Figure S2.

**Figure 7:**
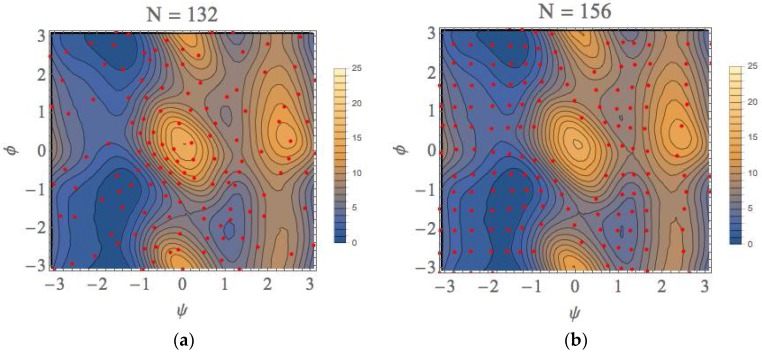
The free-energy landscapes of the (φ, ψ) space for the alanine dipeptide system with using (**a**) our exploration scheme and (**b**) the usual US + WHAM method.

The PMF landscape that was obtained from our scheme and that obtained with the usual umbrella sampling with WHAM (US + WHAM) method are shown in Figure 7a,b, respectively. For the later calculations, we performed the umbrella sampling calculations for the 156 windows at regular intervals with fixing the force constant to 100 kJ/mol/rad^2^ for both of ψ and φ directions. The red points shown in Figure 7b are the average points obtained with the biased runs. We can see from this figure that the free energy landscapes are similar to each other. In fact, the characteristics of all equilibrium states (EQ) and transition states (TS) shown in Table 1 almost completely coincide with each other. The whole computational (wall) times are approximately 58,500 and 70,200 s with intel Core i7 3960X (6 core) processor, respectively, for our method and US + WHAM method (details will be presented in Table S1).

Here, we would like to emphasize that once the local landscapes in the conformational space have been calculated, the characteristics of these landscapes are similar to those obtained after the completion of our procedure (N = 132 in this case: see Figure 7a) even if the calculation is truncated. As shown in Figure S3, the convergences were attained within 0.2 kcal/mol. This is because we utilize the automated sampling scheme to prefer the lower TSs and EQs based on the gradients of local free-energy landscapes. The considerable accuracy of the local landscapes in the truncated calculation results implies that our approach can be utilized to find equilibrium and transition state structures along minimum free-energy paths sequentially without calculating the whole free-energy landscape; because of that, our approach could be most effective in the applications to chemical reaction when the geometry of reactant is known. In particular, this effectiveness will be more remarkable as the dimension of the reaction space increases. 

## 4. Discussion and Future Directions

In this study, we formulated and implemented a new automated exploration scheme of free energy landscapes based on the umbrella integration. With this scheme, we can explore equilibrium conformations (EQ) and transition states (TS) in the ascending order of mean force (PMF) values. We applied our scheme to the free energy landscape for the two backbone torsion angles of the alanine dipeptide. It was demonstrated that the EQs and TSs were practically found in the ascending order of the PMF value and that, even if the iteration procedure was truncated, the PMF landscapes around the EQs and TSs are similar to the completed landscape obtained by our scheme and that obtained by the usual umbrella sampling with WHAM procedure.

Although we only present a fundamental algorithm and a simple application of our method in this study, we intend to apply our method to high-dimensional problems in the future. One might argue that there might be problems in our algorithm for high-dimensional cases. The first point is how to determine candidate points, shown in Figure 2 for the 2D case, for the many-dimensional in general. For this issue, the polar coordinate interpolation technique for a spherical hypersurface proposed by Maeda and Ohno [37] can be used. In this method, they first choose acceptable *f*-dimensional vectors consisting of integers {K*_i_*}, i.e., {(K_1_, K_2_, ..., K*_f_*)}, that satisfy $\overset{f}{\underset{i=1}{\sum }}\left|K_{i}\right|\leq n+M$ where n is the number of non-zero elements in the vector and *M* is an integral parameter (*M* > 0). Then, they map the vectors to the hypersphere to determine the angles of the points. See ref. [37] for details. The application of the method to our ellipse hypersurface can be straightforwardly implemented by replacing the metric by the Mahalanobic metric. The second point is the computational costs for the high dimensional problems. For this point, we would like to emphasize that our method is based on the umbrella integration method, which is critically different from the self-learning umbrella sampling method [14]. Thus, our method is more suitable for finding the low-lying free-energy paths in the reactive coordinate space, not the whole region in the reactive space, by exploiting the gradients (and Hessians) on the PMF surface. The slight changes of our algorithm adapted to the high-dimensional (four and six dimensional) cases, and the computational results, together with comparison with those obtained by the other methods [13,14], will be presented in the near future. 

Finally, we would also emphasize that our approach can be applied not only to all-atoms MD simulations, but also to multiscale simulations [38] such as quantum mechanics/molecular mechanics (QM/MM) MD and all-atoms MD/coarse-grained MD calculations. This would be important when the target reaction coordinate involves the remarkable changes of phases of electronic structures and/or the forming or dissociating of a chemical bond, which often occur in most interesting reaction coordinates in nano-materials and bio-chemical reactions. 

## Figures and Tables

**Table 1:** The characteristics of all equilibrium conformations (EQ) and transition states (TS) calculated with our scheme (N = 132). The values obtained from usual US + WHAM calculations are also listed in parentheses.

	φ (rad)	ψ (rad)	A (kcal/mol)
EQ1 (α_R_)	−1.4 (−1.4)	−1.1 (−1.0)	0.0 (0.0)
EQ2 (C_7_^eq^)	−1.5 (−1.5)	2.9 (2.9)	0.0 (0.0)
EQ3 (C_7_^ex^)	1.1 (1.1)	−2.1 (−2.1)	4.0 (4.0)
EQ4 (α_L_)	1.1 (1.1)	0.9 (0.8)	7.0 (7.0)
TS1	−1.7 (−1.7)	0.6 (0.6)	3.4 (3.4)
TS2	0.2 (0.2)	−1.6 (−1.6)	6.0 (6.0)
TS3	0.2 (0.3)	1.5 (1.4)	8.3 (8.3)
TS4	1.3 (1.3)	−0.1 (−0.1)	8.0 (8.0)

## Supplementary Materials

Supplementary materials can be found at http://www.mdpi.com/1422-0067/19/4/937/s1.

## Abbreviations

PMF	Potential of mean force
US	Umbrella sampling
WHAM	Weighted histogram analysis
UI	Umbrella integration
N.N.	Nearest neighboring
EQ	Equilibrium state
TS	Transition state

## Appendix A

After we determined the child point in the reactive coordinate space, we will next run a simulation with a bias potential for the child window that lies in the neighborhood of the child point. However, it is obvious that the deviation between the average point of the simulation for the child window and the child point strongly depends on the force constant matrix of the biased potential given by Equation (6). In this appendix, we describe how to adjust the bias parameter matrix **K*_c_*** for the child window and the deviation between the child point and the average point for the child window.

First, the gradient of the biased free energy for the child window would be expressed by

(A1) $$g_{c}^{b}\left(\xi \right)=g_{c}^{u}\left(\xi \right)+\omega_{c}\left(\xi \right)=g_{c}^{u}\left(\xi \right)+K_{c}\left(\xi -\xi_{c}^{ref}\right)$$

where $\xi_{c}^{ref}$ and **K*_c_*** are the center and the force constant matrix of the biased harmonic potential for the child window, respectively. On the basis of the fact that the child point is adjacent to the parent point, we assume that the unbiased gradient at the child window is similar to that at the parent window, so that the former coincides with the latter by the translation operation

(A2) $$g_{c}^{u}\left(\xi \right)=g_{p}^{u}\left(\xi -(\xi_{c}^{}-\xi_{p}^{})\right)=g_{p}^{u}\left(\xi -\Delta_{cp}\right).$$

Here $\xi_{c}^{}$ and $\xi_{p}^{}$ are the child and parent points, respectively. Substituting Equation (A2) into Equation (A1), we have

(A3) $$g_{c}^{b}\left(\xi \right)=g_{p}^{u}\left(\xi -\Delta_{cp}\right)+K_{c}\left(\xi -\xi_{c}^{ref}\right).$$

Assuming that the gradient of the biased force is considerably dominated by the harmonic oscillator biased potential, we can expect that the following relation approximately holds:

(A4) $$g_{c}^{b}\left({\langle \xi \rangle }_{c}\right)=0.$$

Here ${\langle \xi \rangle }_{c}$ is the average point calculated for the child window. Then we have,

(A5) $$g_{p}^{u}\left({\langle \xi \rangle }_{c}-\Delta_{cp}\right)+K_{c}\left({\langle \xi \rangle }_{c}-\xi_{c}^{ref}\right)=0.$$

Using Equation (8) in the text for the first term at the left side, we obtain

(A6) $$C_{p}^{-1}\frac{1}{\beta }\left({\langle \xi \rangle }_{c}-\Delta_{cp}-{\langle \xi \rangle }_{p}\right)-K_{p}\left({\langle \xi \rangle }_{c}-\Delta_{cp}-\xi_{p}^{ref}\right)+K_{c}\left({\langle \xi \rangle }_{c}-\xi_{c}^{ref}\right)=0.$$

After some manipulation, the difference between $\xi_{c}^{}$ and ${\langle \xi \rangle }_{c}$ is given by

(A7) $$\Delta_{c}\equiv {\langle \xi \rangle }_{c}-\xi_{c}^{}=\frac{K_{p}\left(\xi_{c}^{ref}-\xi_{p}^{ref}-\Delta_{cp}\right)-C_{p}^{-1}\beta_{}^{-1}\left(\xi_{c}^{ref}-{\langle \xi \rangle }_{p}-\Delta_{cp}\right)}{C_{p}^{-1}\beta_{}^{-1}-K_{p}+K_{c}}+\xi_{c}^{}-\xi_{c}^{ref}.$$

If we intend to attain $\Delta_{c}≅0$ with varying $K_{c}$ and $\xi_{c}^{ref}$, the trivial solution is easily obtained as,

(A8) $$K_{c}=\infty$$

and

(A9) $$\xi_{c}^{ref}=\xi_{c}^{}.$$

However, Equation (A8) implies that the distribution obtained from a simulation with using such an infinite bias potential becomes the delta function, $\delta \left(\xi -\xi_{c}^{ref}\right)$, implying that the coordinate is fixed for the biased calculation. This type of the bias potentials is actually used in the Blue Moon sampling method [16,17], but is not appropriate for the umbrella integration method. In contrast, if the force constant reduces to zero ($K_{c}\sim 0)$, the simulation run will be of the plain molecular dynamics without any bias potential. What we would like to obtain is the reasonable solution for $K_{c}$ and $\xi_{c}^{ref}-\xi_{c}^{}$ which make an automated exploration efficiently work.

To this end, we performed an iteration procedure as follows. First, assuming that (A9) holds, we have

(A10) $${\Delta_{c}^{}|}_{i=0}=\frac{K_{p}\left(\xi_{c}^{ref}-\xi_{p}^{ref}-\Delta_{cp}\right)\;-\;C_{p}^{-1}\beta_{}^{-1}\left(\xi_{c}^{ref}\;-\;{\langle \xi \rangle }_{p}\;-\;\Delta_{cp}\right)}{C_{p}^{-1}\beta_{}^{-1\;}-\;K_{p\;}+\;K_{c}},$$

where the subscript for $\Delta_{c}^{}$ is the index of the iteration procedure. Then we set a threshold, t, for the Mahalanobis distance of $\Delta_{c}^{}$. To define the Mahalanobis distance, we employ the normal distribution function obtained for the parent window. Thus we denote the Mahalanobis distance of $\Delta_{c}^{}$ by ${\left|\Delta_{c}^{}\right|}_{p}$. Then we find a force constant matrix of the diagonal form,

(A11) $$K_{c}=\left[\begin{array}{ccc} k_{1} & 0 & \ldots \\ 0 & k_{2} & \\ \vdots & & \ddots \end{array}\right],$$

which satisfies

(A12) $${\left|{\Delta_{c}^{}|}_{i=0}\right|}_{p}\leq t$$

with minimizing $\overset{}{\underset{j}{\sum }}k_{j}^{2}$ under the condition $30.0\leq \overset{}{\underset{j}{\sum }}k_{j}^{2}$ simultaneously. In the actual calculations shown in this study, t is set to be 0.01. In addition, because we do not prefer either the extremely strong bias or nearly zero bias, we minimize $\;\overset{}{\underset{j}{\sum }}k_{j}^{2}$ with imposing the condition $30.0\leq \overset{}{\underset{j}{\sum }}k_{j}^{2}$. The value of the threshold, t = 0.01, and the lower limit, 30.0, of $\overset{}{\underset{j}{\sum }}k_{j}^{2}\;$ were determined by some test calculations. Once ${\Delta_{c}^{}|}_{i=0}$ is obtained, $\xi_{c}^{ref}$ is shifted by -${\Delta_{c}^{}|}_{i=0}$. We estimate Equation (A7) to update $\Delta_{c}^{}$ to ${\Delta_{c}^{}|}_{i=1}$, as well as ${\langle \xi \rangle }_{c}\equiv \Delta_{c}+\xi_{c}^{}$. Using Equation (A7), we find $K_{c}$ that satisfies

(A13) $${\left|{\Delta_{c}^{}|}_{i=1}\right|}_{p}\leq t$$

under the conditions $\overset{}{\underset{j}{\sum }}k_{j}^{2}$ being minimum and $30.0\leq \overset{}{\underset{j}{\sum }}k_{j}^{2}$. After that, $\xi_{c}^{ref}$ is shifted by -${\Delta_{c}^{}|}_{i=1}$ again. Using the updated value of $\xi_{c}^{ref}$, we estimate ${\left|\xi_{c}^{ref}-{\langle \xi \rangle }_{c}^{}\right|}_{p}$ and if the value is less than a threshold, the computational procedure is complete. Otherwise, go back to Equation (A7). We can summarize the procedure as follows:

which satisfies

(A0) Start with the assumption that $\xi_{c}^{ref}=\xi_{c}^{}$ holds.
(A1) Estimate $\Delta_{c}$, Mahalanobis distance, ${\left|\Delta_{c}^{}\right|}_{p}$, and ${\langle \xi \rangle }_{c}=\Delta_{c}+\xi_{c}^{}$.
(A2) Varying $K_{c}=\left[\begin{array}{cc} k_{x} & 0\\ 0 & k_{y} \end{array}\right]$ for satisfying ${\left|\Delta_{c}^{}\right|}_{p}\leq t$, with minimizing $k_{x}^{2}+k_{y}^{2}$.
(A3) Update $\xi_{c}^{ref}$: $\xi_{c}^{ref}-\Delta_{c}^{}\to \xi_{c}^{ref}$, and go back to (A1).
(A4) Estimate ${\left|\xi_{c}^{ref}-{\langle \xi \rangle }_{c}^{}\right|}_{p}$. If this value is less than a threshold, which is set to be 1.5 for the actual calculations shown in the text.
