# Supplementary material for: Automated Exploration of Free Energy Landscapes Based on Umbrella Integration
Source: Int J Mol Sci. 2018 Mar 21;19(4):937. doi: 10.3390/ijms19040937 (PMC5979611; doi:10.3390/ijms19040937)
Supplement: Supplementary file 1 [file ijms-19-00937-s001.pdf]

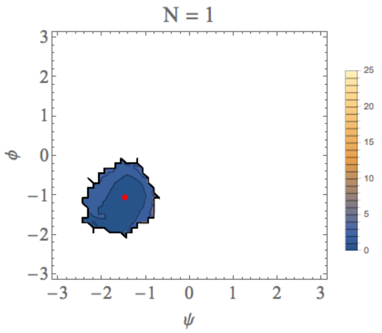

(1) N=1

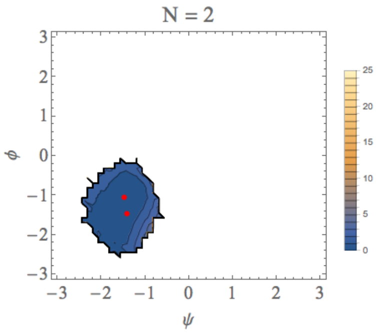

(2) N=2

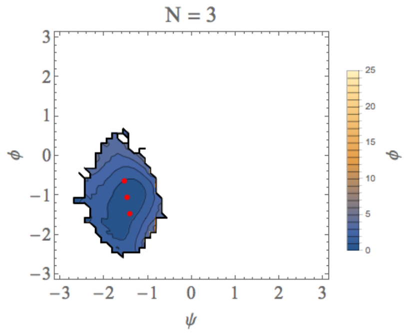

(3) N=3

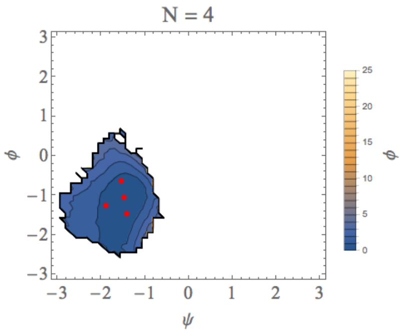

(4) N=4

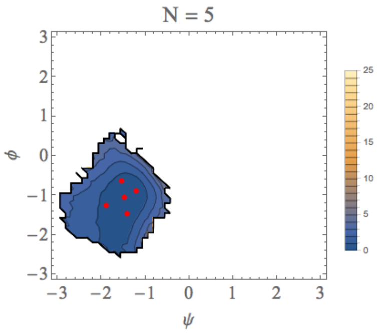

(5) N=5

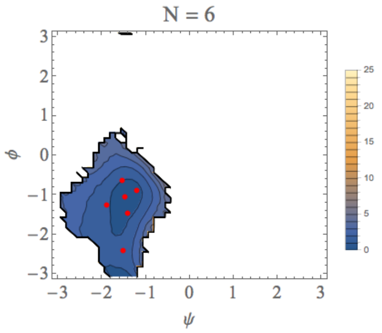

(6) N=6

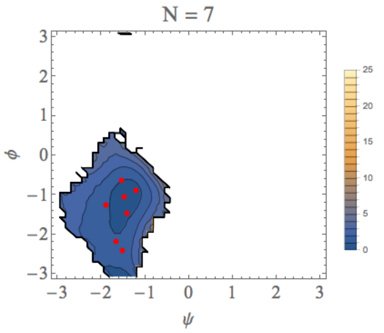

(7) N=7

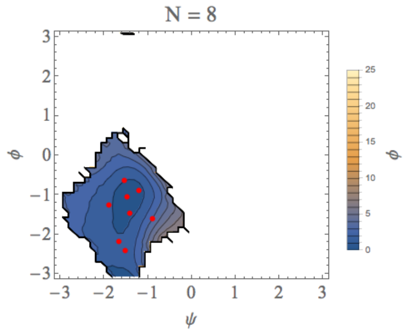

(8) N=8

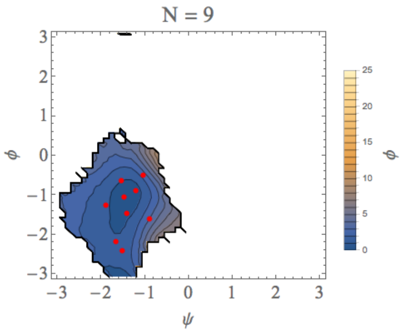

(9) N=9

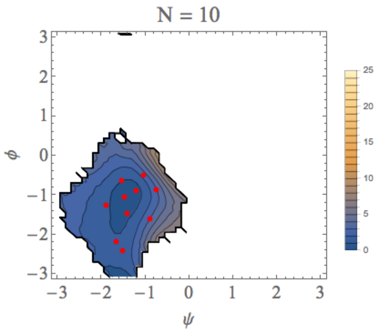

(10) N=10

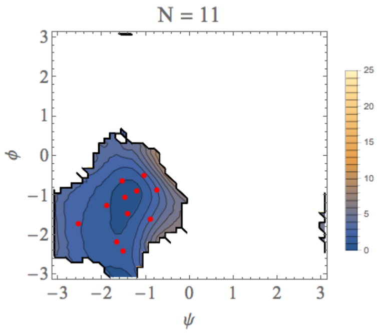

(11) N=11

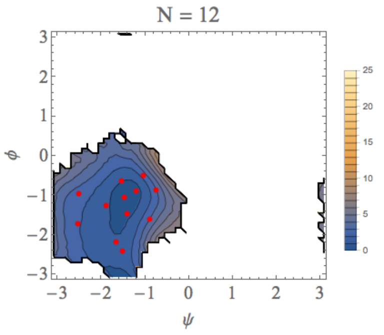

(12) N=12

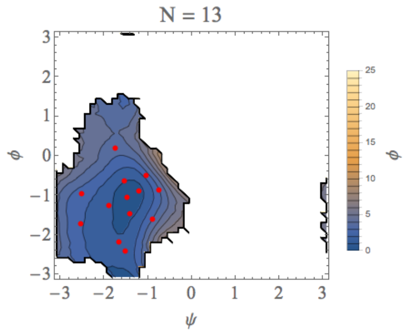

(13) N=13

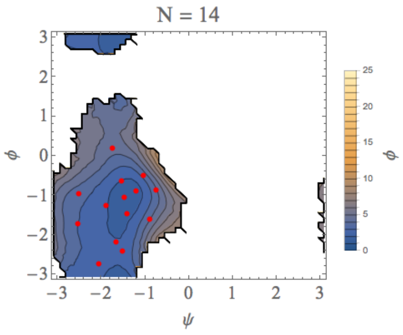

(14) N=14

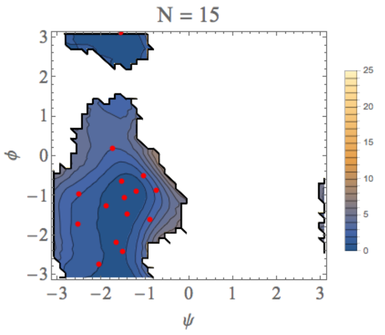

(15) N=15

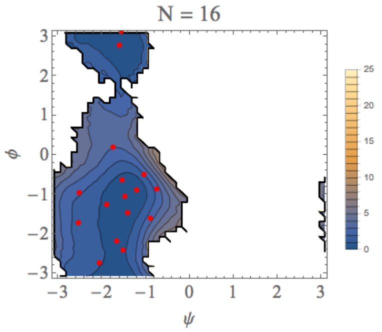

(16) N=16

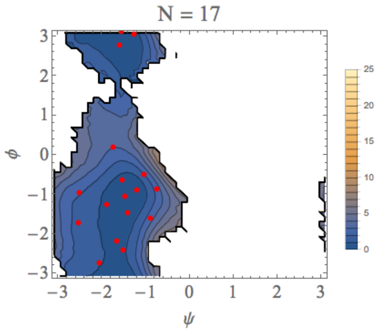

(17) N=17

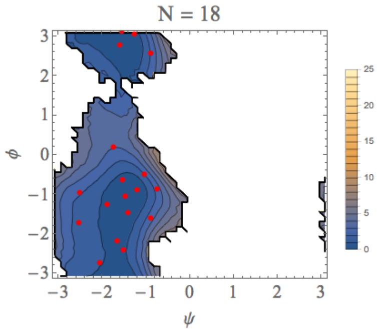

(18) N=18

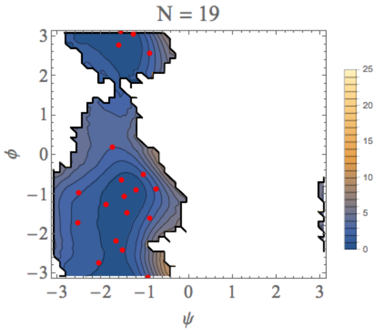

(19) N=19

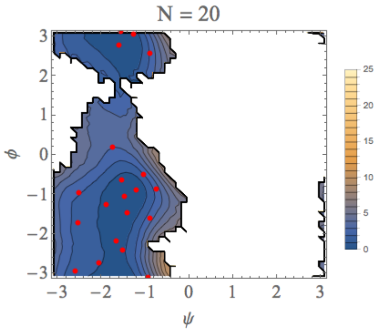

(20) N=20

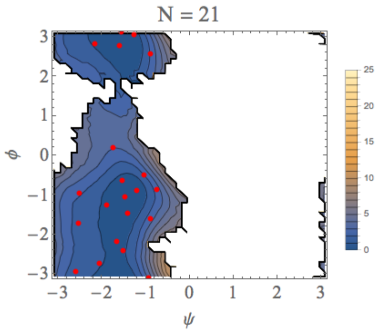

(21) N=21

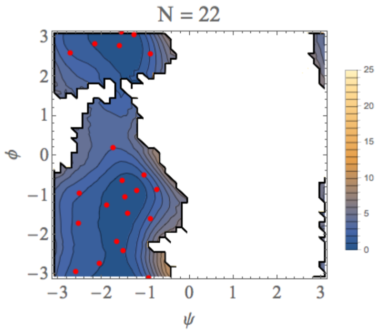

(22) N=22

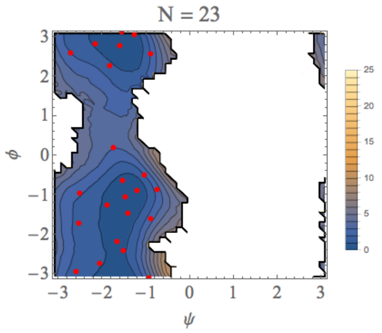

(23) N=23

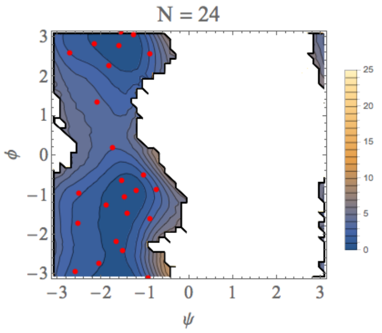

(24) N=24

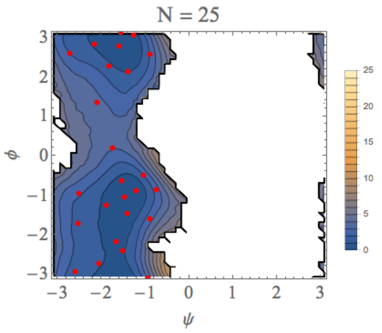

(25) N=25

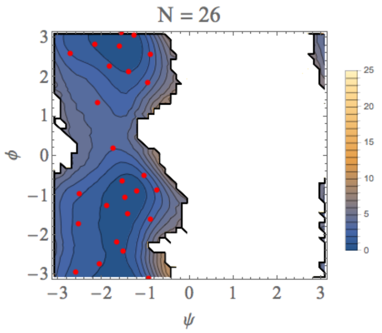

(26) N=26

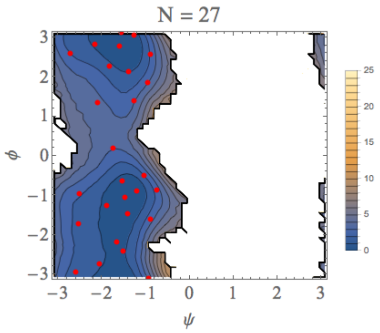

(27) N=27

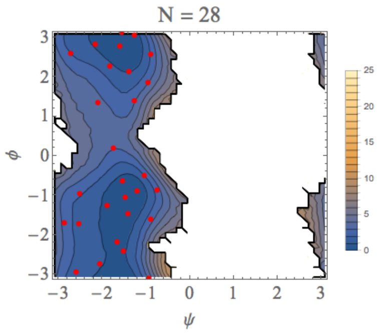

(28) N=28

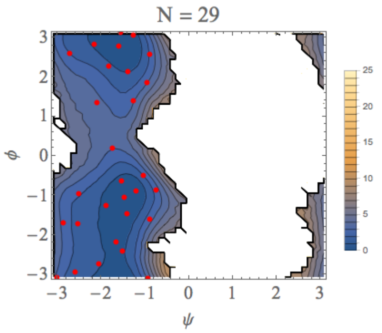

(29) N=29

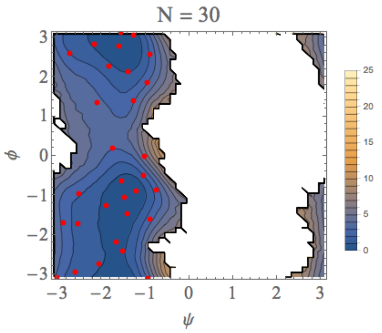

(30) N=30

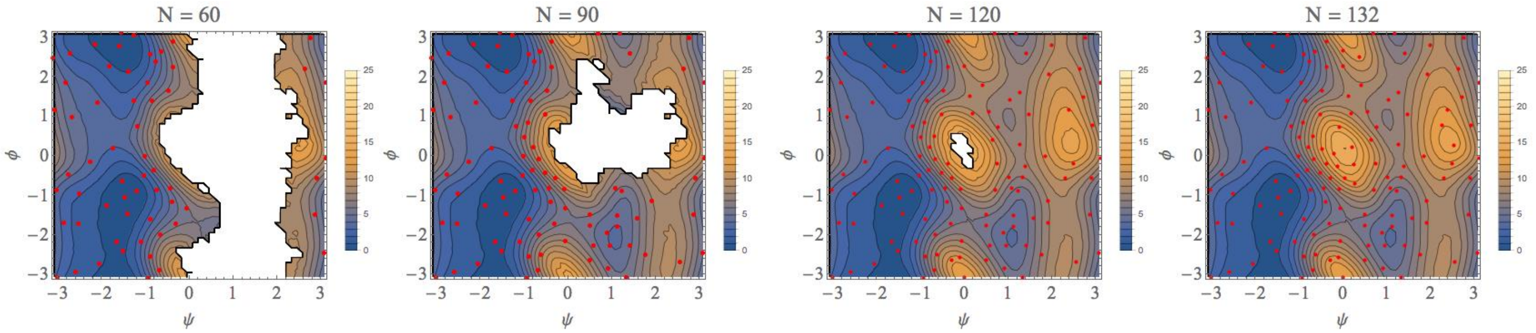

(31) N=60 (32) N=90 (33) N=120 (34) N=132

Figure S1 The free-energy landscapes of the (Φ, Ψ) space for the alanine dipeptide system with using our exploration scheme. N is the number of iterations to obtain each figure. The PMF values at the regions colored in white, where the sampling data are not obtained, are set to infinity.

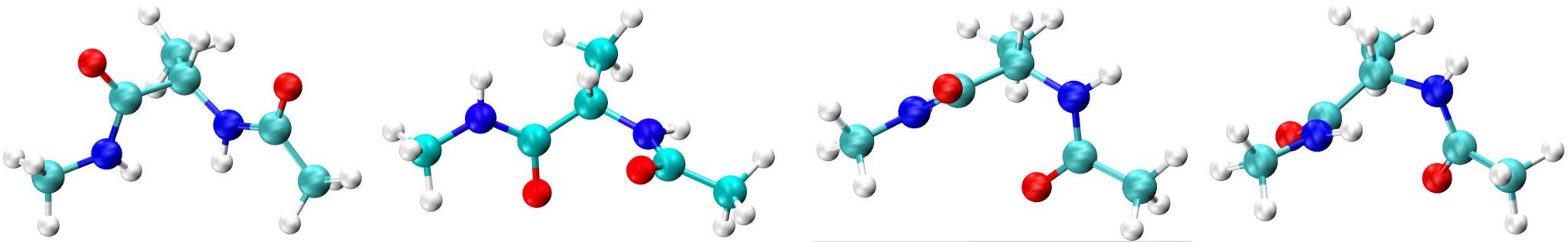

(a) (b) (c) (d)

Figure S2 The typical structures of α_R, C_7^{eq}, C_7^{ex}, and α_L conformations obtained from our automated exploration procedure.

Table S1 Computational (wall and core) times we used for calculations of our method and the US+WHAM method (*).

	Our method	The US+WHAM method
One equilibration process per window (sec)	21 (128)	21 (128)
One run for sampling per window (sec)	429 (2568)	429 (2568)
The whole run (sec)	58500	70200

(*) Intel Core i7 3960X (6 core) processor is used. The core times are also shown in parentheses.

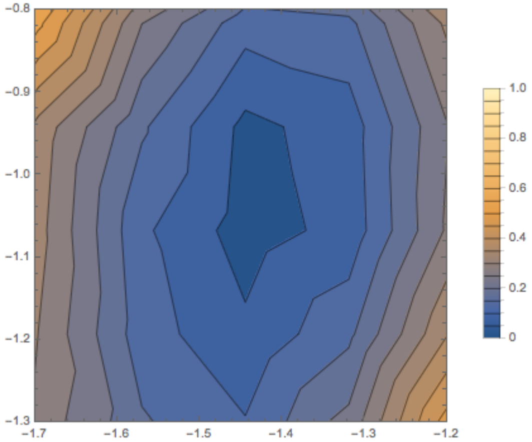

(EQ1) N=60

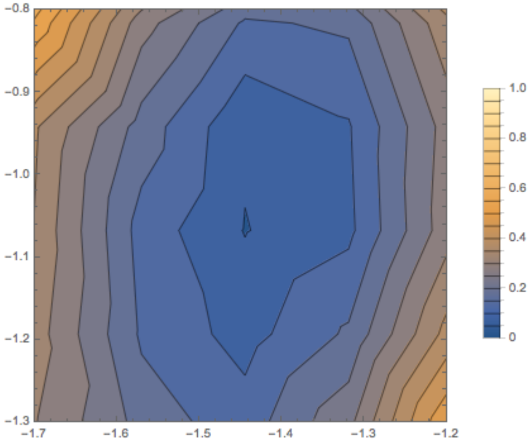

(EQ1) N=90

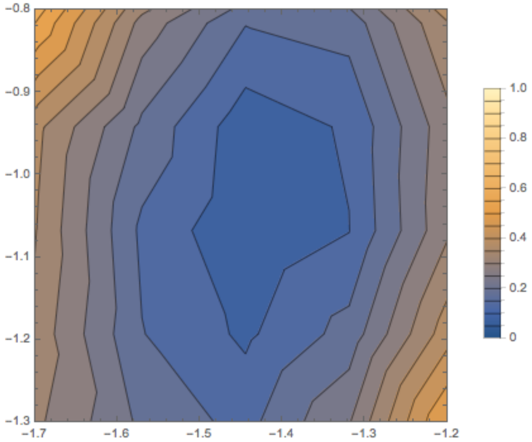

(EQ1) N=132

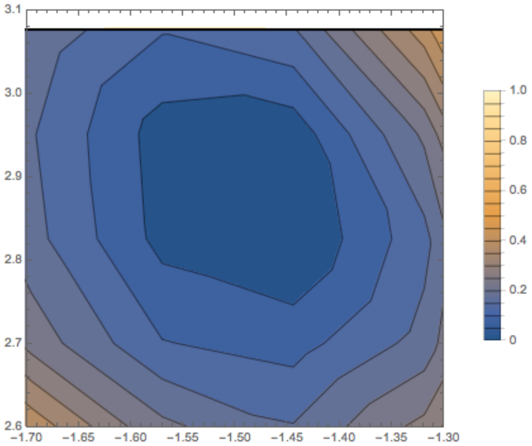

(EQ2) N=60

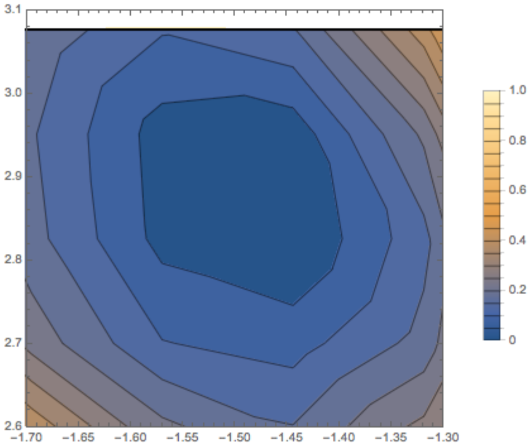

(EQ2) N=90

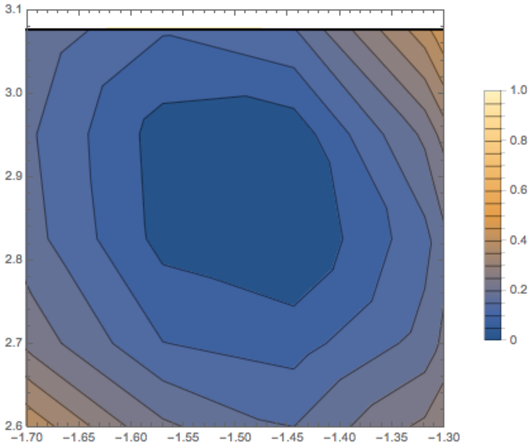

(EQ2) N=132

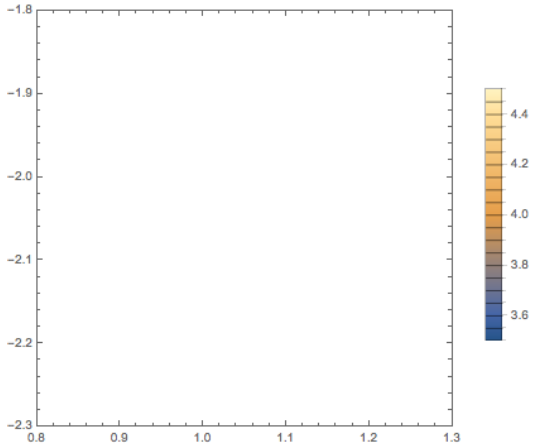

(EQ3) N=60

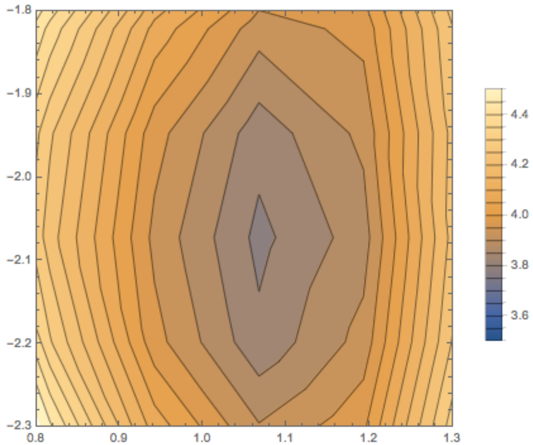

(EQ3) N=90

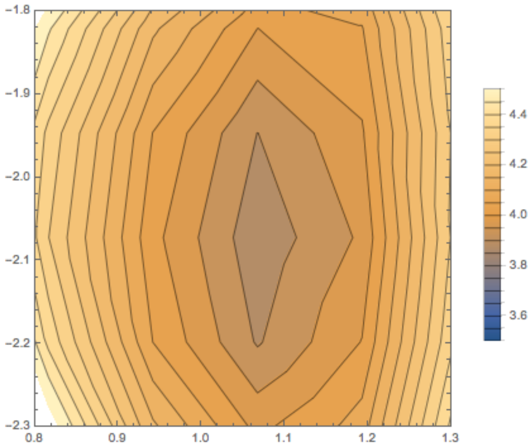

(EQ3) N=132

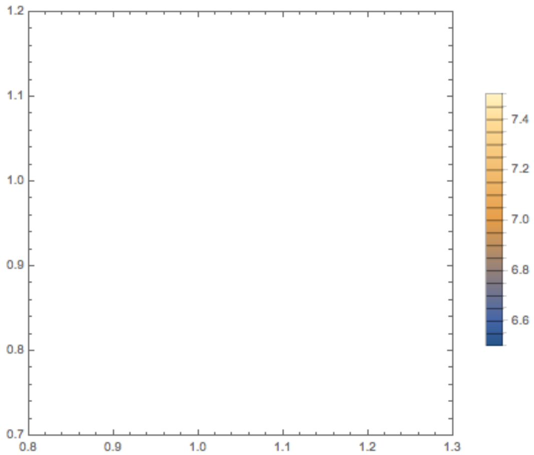

(EQ4) N=60

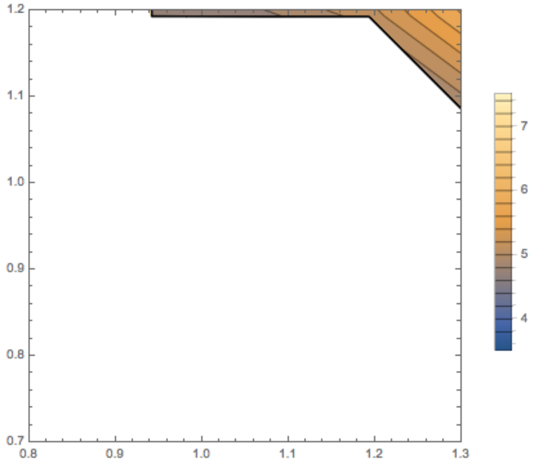

(EQ4) N=90

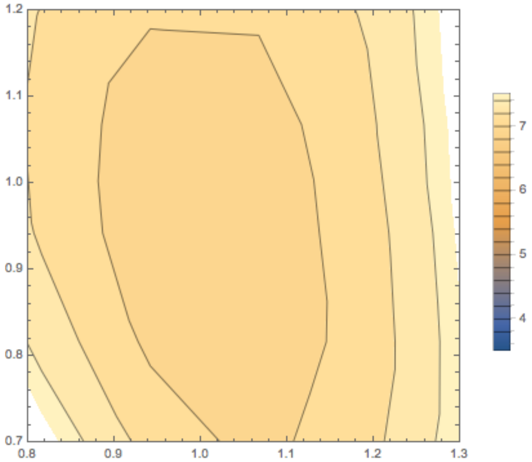

(EQ4) N=132

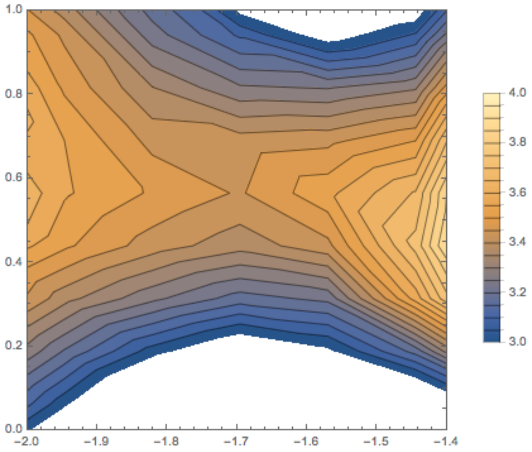

(TS1) N=60

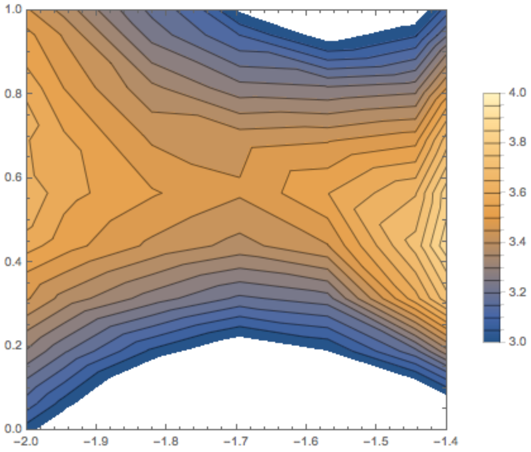

(TS1) N=90

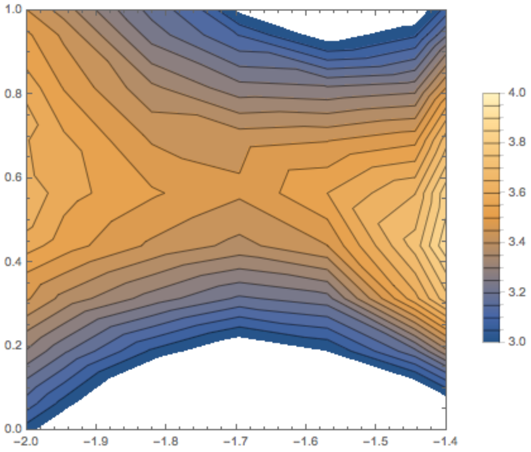

(TS1) N=132

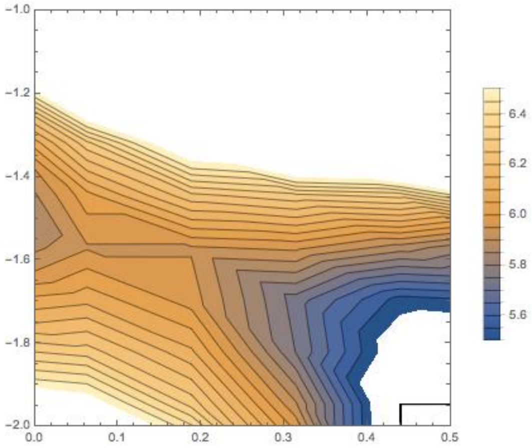

(TS2) N=60

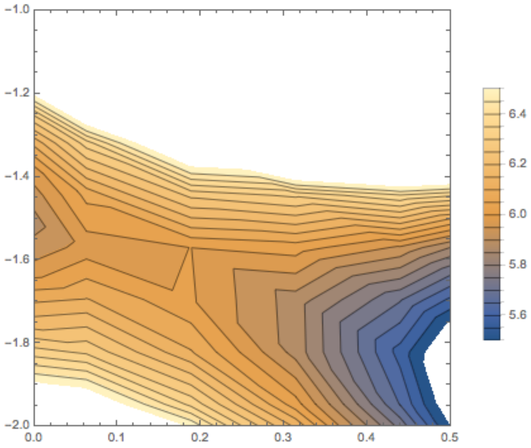

(TS2) N=90

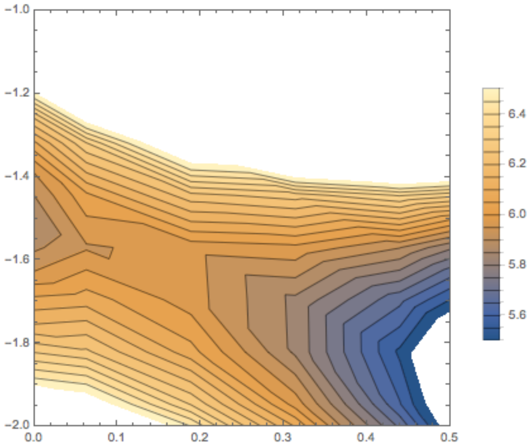

(TS2) N=132

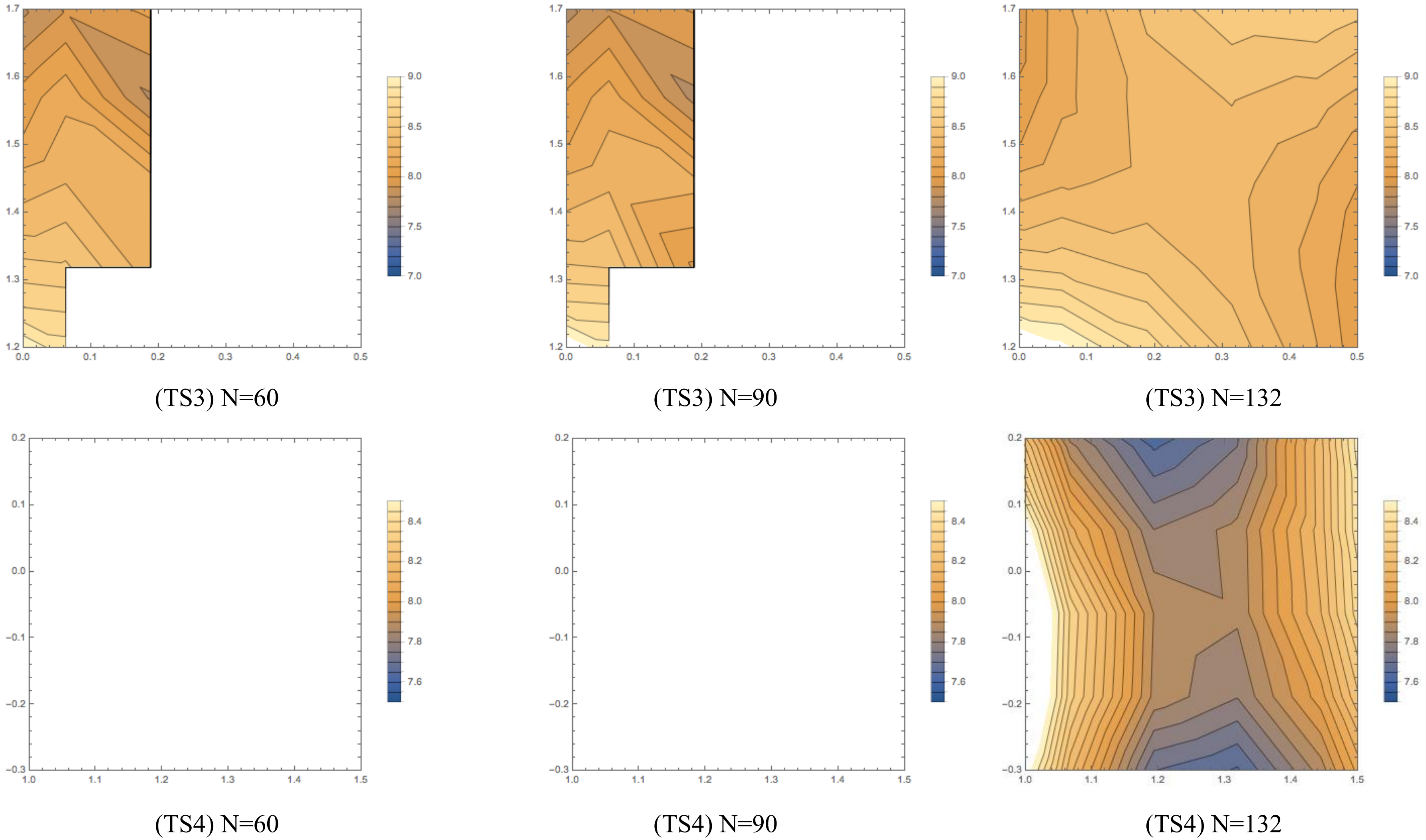

Figure S3 The free-energy landscapes for the local areas around the equilibrium conformations (EQ) and transition states (TS). N is the number of iterations to obtain each figure. EQn (n=1~4) and TSn(1-4) correspond to those presented in table 1. The white landscape corresponds to the area, in which the sampling has not been performed yet (see Figure S1) and so the PMF values were set to infinite for clarity. Because the numerical treatment makes the PMF values of the points surrounded by the white region unreliable, we do not color such points.
